# Interaction of sedentary behaviour and educational level in breast cancer risk

**DOI:** 10.1371/journal.pone.0300349

**Published:** 2024-05-16

**Authors:** Marina Pinto-Carbó, Mercedes Vanaclocha-Espí, Josefa Ibañez, Javier Martín-Pozuelo, Paula Romeo-Cervera, Andreu Nolasco, María Besó-Delgado, Susana Castán-Cameo, Dolores Salas, Ana Molina-Barceló

**Affiliations:** 1 Cancer and Public Health Research Unit, Foundation for the Promotion of Health and Biomedical Research of Valencia Region (FISABIO-Public Health), Valencia, Spain; 2 Ministry of Universal Health and Public Health, Valencia, Spain; 3 Research Unit for the Analysis of Mortality and Health Statistics, Department of Community Nursing, Preventive Medicine, Public Health and History of Science, University of Alicante, Alicante, Spain; 4 Preventive Medicine Service, General Hospital of Requena, Valencia, Spain; 5 General Directorate of Public Health and Addictions, Valencia, Spain; 6 Spanish Consortium for Research on Epidemiology and Public Health (CIBERESP), Health Institute Carlos III, Madrid, Spain; Saga University, JAPAN

## Abstract

**Objective:**

This cross-sectional study aims to analyse the relationship between sedentary behaviour and breast cancer (BC) risk from a social perspective.

**Methods:**

Women aged 45–70 who participated in the Valencia Region Breast Cancer Screening Programme (2018–2019) were included, with a total of 121,359 women analysed, including 506 with cancer and 120,853 without cancer. The response variable was BC (screen-detected) and the main explanatory variable was sedentary behaviour (≤2 / >2-≤3 / >3-≤5 / >5 hours/day, h/d). Nested logistic regression models (M) were estimated: M1: sedentary behaviour adjusted for age and family history of BC; M2: M1 + hormonal/reproductive variables (menopausal status, number of pregnancies, hormone replacement therapy; in addition, months of breastfeeding was added for a subsample of women with one or more live births); M3: M2 + lifestyle variables (body mass index, smoking habits); M4: M3 + socioeconomic variables (educational level, occupation); Final model: M4 + gender variables (childcare responsibilities, family size). Interaction between sedentary behaviour and educational level was analysed in the Final model. Moreover, for the whole sample, postmenopausal women and HR+ BC, the Final model was stratified by educational level.

**Results:**

Sedentary behaviour was associated with an increased risk of BC with a nearly statistically significant effect in the Final model (>2-≤3 h/d: OR = 1.22 (0.93–1.61); >3-≤5 h/d: OR = 1.14 (0.86–1.52); >5: OR = 1.19 (0.89–1.60)). For women with a low educational level, sitting more than 2 h/d was associated with an increased risk of BC in the whole sample (>2-≤3 h/d OR = 1.93 (1.19–3.21); in postmenopausal women (>2-≤3 h/d, OR = 2.12 (1.18–2.96), >5h/d OR = 1.75 (1.01–3.11)) and in HR+ BC (>2-≤3h/d, OR = 2.15 (1.22–3.99)). Similar results were observed for women with one or more live births.

Conclusions

Sitting >2 h/d is associated with BC risk in women with low educational level, especially in postmenopausal women and those with live births.

## Introduction

Breast cancer (BC) is a significant public health problem worldwide [1]. In Spain, it is the most common type of cancer in women and causes the highest number of cancer deaths in this same population group [2].

The high incidence and mortality rate of BC demonstrate the need to investigate its risk factors. Only 5–10% of cancers are caused by genetic factors, and the remaining 90–95% are due to social, lifestyle and environmental aspects [3]. Several studies show that a diet rich in processed meat [4], alcohol consumption [5], smoking [6], and physical inactivity [7] are modifiable BC risk factors.

Most studies that analyse the relationship between BC and physical activity have focused on physical inactivity defined as the failure to meet World Health Organization (WHO) recommendations [8]. However, there is currently little consensus on the relationship between sedentary behaviour that is defined as any activity with an energy expenditure of less than 1.5 metabolic equivalents (METs) such as watching television, or driving [9], and BC. Some studies show a positive relationship between sedentary behaviour and BC risk [10–13], while other studies find no such association [14, 15].

Sedentary behaviour may influence the risk of BC through inflammatory, metabolic, and hormonal processes. It has been observed that sedentary behaviour leads to increased blood insulin level, which is associated with an increase of BC risk [9, 16, 17]. In addition, it has been proven that the bioavailability of free oestrogens (oestradiol, free oestradiol, and oestrone), which are released by adipose tissue in postmenopausal women [17], can induce cellular proliferation and stimulate tumour growth [16, 17]. Considering this, sedentary behaviour has been shown to increase both triglycerides and cholesterol in the blood [9], which may increase adipose tissue in women and consequently, the release of these free oestrogens.

There is strong evidence that postmenopausal status [18, 19], nulliparity [20, 21], increased age at first birth [22] and the use of hormone replacement therapy (HRT) [23] are common BC risk factors. It is also widely known that breastfeeding plays a protective role against this type of cancer, with the longer breastfeeding lasts, the greater the reduction in risk [10]. There is no consensus regarding the relationship between sedentary behaviour and BC risk according to menopausal status. Some studies observed that sedentary behaviour has a more significant impact on BC risk in postmenopausal women [18, 19], while others do not observe this difference [5, 12]. The same is true in the impact according to different hormone receptor subtype [11], with some studies observing that sedentary behaviour has a higher impact on hormone receptor-positive (HR+) BC [19].

It is known that smoking and high body mass index (BMI) increase the risk of BC [24–26]. The impact of these factors on BC when combined with sedentary behaviour is currently unknown. Although there is no consensus regarding the direction of the relationship between sedentary behaviour and BMI, as sedentary behaviour may entail an increase in BMI and the amount of adipose tissue, in combination they may both increase BC risk.

Likewise, we know that women with a high educational level are more likely to develop BC [27–29]. This may be due to the presence of hormonal and reproductive risk factors for BC associated with this group, such as nulliparity and HRT use [30, 31]. Moreover, it has been observed that women with high educational level have a higher risk of sedentary behaviour than those with a low educational level [32]. Although the influence of educational level on BC risk and sedentary behaviour has been observed, to the authors’ knowledge, there are no studies that analyse the relationship between sedentary behaviour and BC while considering this social determinant. As regards occupation, there is currently little consensus on its relationship with BC. While some studies show that non-manual occupations increase BC risk [33, 34], others find no such association [14]. Moreover, there are currently no studies that include gender factors, such as women’s family caregiving responsibilities, in BC risk analysis.

Due to these controversial results regarding the association between sedentary behaviour and BC and the lack of studies including the influence of social and gender determinants on this association, the objective of this study is to analyse the relationship between sedentary behaviour and BC from a social perspective.

## Materials and methods

### Study population

This cross-sectional study includes all women aged between 45 and 70 who participated in the population-based Valencia Region Breast Cancer Screening Programme (VR-BCSP) (Spain) between November 2018 and October 2019 (N = 289,315). The sample analysed were women who had concluded the screening process and for whom there was data on sedentary behaviour available (n = 121,359). A total of 506 women with screen-detected BC and 120,853 women without cancer were analysed.

### Information collection

The data analysed were obtained from the VR-BCSP information system. This system provides information on the results of the screening process and, in the event of a BC diagnosis, on tumour characteristics. It also includes socioeconomic and lifestyle data. This information is collected through an epidemiological questionnaire that is conducted face to face with women when they attend their biennial programme appointment and before a possible BC diagnosis. Information on sedentary behaviour was collected using the short version of the International Physical Activity Questionnaire (IPAQ-short), which is part of the epidemiological questionnaire. This IPAQ-Short questionnaire has been internationally validated for collecting information on physical activity and sedentary behaviour [35].

In addition, the information system includes the Segmented, Integrated, and Geographic Population Analysis Code, which is obtained by linking data with the Population Information System of the Valencia Region Government’s Department of Universal and Public Health. This code collects individual information on variables related to socioeconomic and gender characteristics for the entire population with the right to healthcare coverage, including family size or educational level.

### Ethics considerations

This study was approved by the Research Ethics Committee of the General Directorate of Public Health and the Advanced Public Health Research Centre (CEI DGSP-CSISP) on 9 January 2,020 (reference no.: 20200109/04). Taking into account the project design and its large sample size, the ethics committee approved carrying out the study without requiring that individual consent be provided by each subject, in accordance with the regulations of the current version of the Declaration of Helsinki (October 2008, Seoul). The personal data included in this study was pseudonymised to guarantee confidentiality, privacy, and security. The project was carried out in accordance with the principles of the Declaration of Helsinki and Spanish confidentiality legislation (Spanish Organic Law 3/2018, of 5 December, on Personal Data Protection and Guaranteeing Digital Rights).

### Study variables

The response variable is screen-detected BC (yes/no). In the context of the VR-BCSP, BC is defined as any tumour considered to be a breast carcinoma following a positive screening exam (mammogram) that is confirmed by pathological anatomy. Lobular carcinoma in situ is excluded from this definition. In terms of phenotype, BC is classified as HR+ (oestrogen receptor-positive, and/or progesterone receptor-positive and HER2 negative), Human Epidermal growth factor Receptor 2 (HER2+) (HER2+ regardless of ER or PR expression), or triple negative (TN) (oestrogen receptor-negative, progesterone receptor-negative and HER2 negative).

The main explanatory variable is sedentary behaviour, which was determined by the question “During the last 7 days, how much time did you spend sitting on a week day?” from the IPAQ-short questionnaire (available on https://sites.google.com/view/ipaq?pli=1). In accordance with the questionnaire protocol, the variable is classified based on the interquartile ranges of the sitting time: ≤2 / >2-≤3 / >3-≤5 / >5 hours/day sitting.

Based on scientific evidence, the following variables were considered:

*Adjustment*: age (continuous variable) and family history of BC (yes/no). The “yes” category of the family history variable includes: very strong (first-degree relative with BC in premenopausal years or bilateral cancer), strong (first-degree relative with BC in postmenopausal years or unilateral cancer), and minor (second-degree or more distant relative).
*Hormonal/reproductive*: menopausal status (premenopausal/postmenopausal), months of breastfeeding (0/<6/≥6), number of pregnancies (0/1/≥2), and HRT use (yes/no). The number of pregnancies variable refers to all the pregnancies that the women have had regardless of duration and outcome.
*Lifestyle*: BMI (as a continuous variable) calculated based on self-reported weight (Kg) divided by self-reported height squared (m^2^) and smoking habits (non-smoker/former /current).
*Socioeconomic*: educational level (low/medium-high) and occupation (non-manual/manual/not working/homemaker). The “low” educational level includes illiterate, no schooling, and primary education only, and the “medium-high” category comprises secondary education, post-secondary non tertiary and tertiary education.
*Gender*: childcare responsibilities (yes/no) and family size (FS) (small/medium-large). A small FS is a family unit of <3 people, including women who live alone.

### Statistical analysis

The relationship between BC and sedentary behaviour and the set of explanatory variables was assessed by performing a bivariate analysis through a chi-square or Mann-Whitney U test.

Next, nested logistic regression models (M) were estimated to analyse the relationship between BC risk and sedentary behaviour considering other explanatory variables. The models analysed were: M1: BC risk associated with sedentary behaviour adjusted for age and family history of BC; M2: M1 and hormonal/reproductive variables; M3: M2 and lifestyle variables; M4: M3 and socioeconomic variables; and then, the Final model: M4 and gender variables.

Multivariate logistic regression models were used to explore the possible relationship between sedentary behaviour and BC risk. Moreover, to analyse the interaction between sedentary behaviour and educational level (as categorical variables), this interaction was added to the Final model, which includes all the explanatory variables. This interaction has been explored for the whole sample and according to menopausal status and for the BC phenotypes: HR+ (HR+ vs. no cancer) and HER2+ (HER2+ vs. no cancer). The TN phenotype was not included in the analysis as the sample size was insufficient (TN = 20). For the models where the interaction was statistically significant, stratification by educational level has been analysed.

In order to include the months of breastfeeding variable in the analysis, the same statistical analysis explained above were conducted for a subsample of women with one or more live births (n = 108,121).

The models results are presented in terms of odds ratio (OR) and 95% confidence interval (CI), considering a significance level of less than 0.05. The statistical programme R v4.1.1 was used.

## Results

According to the results of the descriptive analysis (Table 1), >2-≤3 h/d of sitting is the most frequent sedentary behaviour category in both women with BC and without BC (29.45% and 26.58% respectively, p = 0.197). A statistically significant association can be seen between BC and increasing age (p<0.001), having a family history of BC (p = 0.047), number of pregnancies (p = 0.002), months of breastfeeding (p = 0.026), higher BMI (p<0.001), smoking habits (p = 0.021), childcare responsibilities (p = 0.005) and FS (p = 0.005).

**Table 1:** Characteristics of women with and without breast cancer from the sample.

	**Cancer**	**No cancer**	**p value**
	**n (%)**	**n (%)**	
	506	120,853	
**Sedentary behaviour (h/d sitting)**			
≤2	109 (21.54)	30,666 (25.37)	0.197
>2-≤3	149 (29.45)	32,126 (26.58)	
>3-≤5	126 (24.90)	28,909 (23.92)	
>5	122 (24.11)	29,152 (24.12)	
**Age (years)** ^**a**^ **median (min-max)**	57.5 (45–70)	56.0 (45–70)	<0.001
**Family history of BC**			
No	356 (70.50)	89,582 (74.36)	0.047
Yes	149 (29.50)	30,888 (25.64)	
**Hormonal/Reproductive variables**			
**Menopausal status**			
Premenopausal	140 (28.40)	36,635 (31.23)	0.175
Postmenopausal	353(71.60)	80,668 (68.77)	
**Number of pregnancies**			
≥2	333 (66.07)	88,037 (72.97)	0.002
1	102 (20.24)	19,649 (16.29)	
None	69 (13.69)	12,959 (10.74)	
**Months of breastfeeding** ^**b**^			
≥6	183 (43.57)	50,618 (48.90)	0.026
<6	115 (27.38)	28,654 (27.68)	
0	122 (29.05)	24,237 (23.42)	
**HRT**			
No	465 (91.90)	112,933 (93.45)	0.160
Yes	41 (8.10)	7,920 (6.55)	
**Lifestyle variables**			
**BMI** ^**a**^ **median (min-max)**	2,711 (16.85–47.26)	2,635 (14.17–57.78)	<0.001
**Smoking habits**			
Non-smoker	238 (47.04)	62,362 (51.60)	0.021
Current	133 (26.28)	32,225 (26.66)	
Former	135 (26.68)	26,266 (21.73)	
**Gender variables**			
**Childcare responsibilities**			
No	100 (19.92)	30,269 (25.40)	0.005
Yes	402 (80.08)	88,888 (74.60)	
**FS**			
Small	213 (42.43)	43,315 (36.35)	0.005
Medium-Large	289 (57.57)	75,842 (63.65)	
**Socioeconomic variables**			
**Educational level**			
Low	164 (35.04)	34,814 (31.37)	0.097
Medium-High	304 (64.96)	76,153 (68.63)	
**Occupation**			
Manual	132 (28.70)	34,670 (31.41)	0.271
Non-manual	107 (23.26)	27,501 (24.92)	
Not working	50 (10.87)	11,654 (10.56)	
Homemakers	171 (37.17)	36,539 (33.11)	
**Tumour characteristics** ^**c**^			
HR+	323 (81.57)	-	
HER2+	53 (13.38)	-	
TN	20 (5.05)	-	

^a^Mann-Whitney U test

^b^For the subsample of women with one or more live births

^c^ % calculated from the total cases with information on their phenotype (n = 396)

Abbreviations: h/d (hours per day); BC (breast cancer); HRT (Hormone Replacement Therapy); BMI (Body Mass Index); FS (Family Size); HR+ (oestrogen receptor-positive, and/or progesterone receptor-positive and HER2 negative), HER2+ (HER2+ regardless of ER or PR expression), TN (Triple Negative -oestrogen receptor-negative, progesterone receptor-negative and HER2 negative-)

The nested logistic regression models (Table 2) show that sedentary behaviour is positively associated with BC risk. In Model 1, which includes age and family history of BC as adjustment variables, sitting >2-≤3h/d shows a statistically significant relationship (OR = 1.29 (1.01–1.66), p-value = 0.044), being the rest of categories nearly significant (>3-≤5 h/d: OR = 1.18 (0.91–1.53), p-value = 0.206; >5 h/d: OR = 1.21 (0.93–1.57), p-value = 0.156). When including hormonal/reproductive (M2), lifestyle (M3), socioeconomic (M4), and gender (Final model) variables, similar results are observed. In the Final model, the OR for sitting >2-≤3 h/d is 1.22 (CI = 0.93–1.61, p-value = 0.161); for >3-≤5 h/d is 1.14 (CI = 0.86–1.52, p-value = 0.360) and for >5 h/d is 1.19 (CI = 0.89–1.60, p-value = 0.231).

**Table 2:** Relationship between sedentary behaviour and breast cancer risk adjusting for additional explanatory variables. Nested logistic regression models (M) for the whole sample.

	Sedentary behaviour (h/d sitting)	OR (CI)	p value
**Model 1**: sedentary behaviour + age and family history of BC	≤2	1	
	>2-≤3	1.29 (1.01–1.66)	0.044
	>3-≤5	1.18 (0.91–1.53)	0.206
	>5	1.21 (0.93–1.57)	0.156
**Model 2**: M1 + hormonal/reproductive variables (menopausal status, number of pregnancies, HRT)	≤2	1	
	>2-≤3	1.26 (0.98–1.62)	0.071
	>3-≤5	1.17 (0.90–1.52)	0.025
	>5	1.13 (0.87–1.48)	0.349
**Model 3**: M2 + lifestyle-related variables (BMI, smoking habits)	≤2	1	
	>2-≤3	1.27 (0.99–1.65)	0.064
	>3-≤5	1.18 (0.90–1.54)	0.228
	>5	1.13 (0.87–1.48)	0.367
**Model 4**: M3 + socioeconomic variables (educational level, occupation)	≤2	1	
	>2-≤3	1.24 (0.94–1.64)	0.130
	>3-≤5	1.14 (0.86–1.52)	0.359
	>5	1.19 (0.89–1.60)	0.229
**Final model**: M4 + gender-related variables (childcare responsibilities, FS)	≤2	1	
	>2-≤3	1.22 (0.93–1.61)	0.161
	>3-≤5	1.14 (0.86–1.52)	0.360
	>5	1.19 (0.89–1.60)	0.231

Abbreviations: h/d (hours per day); BC (breast cancer); HRT (Hormone Replacement Therapy); BMI (Body Mass Index); FS (Family Size)

When studying the interaction between sedentary behaviour and educational level added in the Final model, a statistically significant relationship is observed for the whole sample (p-int = 0.015), in postmenopausal women (p-int = 0.005) and in HR+ BC (p-int = 0.013) (results not shown in table). This is also observed for the subsample of women with one or more live births (whole subsample: p-int = 0.048; postmenopausal women: p-int = 0.013; HR+ BC: p-int = 0.049) (results not shown in table).

Stratifying by educational level, the results for the whole sample show that women with low educational level who spent >2-≤3 h/d sitting are at a higher BC risk (OR = 1.93 (1.19–3.21), p-value = 0.008) with statistically significant differences (Table 3). This relationship is also found in women with low educational level who spend >3-≤5 h/d (OR = 1.51 (0.92–2.54), p-value = 0.113) and >5 h/d sitting (OR = 1.66 (0.98–2.86), p-value = 0.061) with a nearly statistical significance (Table 3). These results are not observed in women with high educational level. Additional information of other explanatory variables results is provided in S1 Table.

**Table 3:** Relationship between sedentary behaviour and breast cancer risk stratified by educational level: Final Model for the whole sample, postmenopausal women and HR+ breast cancer (HR+ vs. no cancer).

			Educational level		
	Sedentary behaviour (h/d sitting)	Low OR (CI)	p value	Medium-High OR (CI)	p value
**Whole sample** ^a^					
	≤2	1		1	
	>2-≤3	1.93 (1.19–3.21)	0.008	0.96 (0.68–1.35)	0.803
	>3-≤5	1.51 (0.92–2.54)	0.113	1.00 (0.70–1.43)	0.986
	>5	1.66 (0.98–2.86)	0.061	1.01 (0.71–1.44)	0.952
**Postmenopausal women** ^b^					
	≤2	1		1	
	>2-≤3	2.12 (1.28–3.62)	0.004	0.84 (0.55–1.29)	0.434
	>3-≤5	1.63 (0.97–2.83)	0.070	0.90 (0.59–1.39)	0.640
	>5	1.75 (1.01–3.11)	0.049	0.84 (0.54–1.31)	0.435
**HR+ BC** ^c^					
	≤2	1		1	
	>2-≤3	2.15 (1.22–3.99)	0.011	0.89 (0.58–1.36)	0.601
	>3-≤5	1.45 (0.79–2.75)	0.242	0.89 (0.57–1.37)	0.587
	>5	1.60 (0.84–3.12)	0.156	0.74 (0.47–1.17)	0.206

^a^Final model variables: sedentary behaviour, age, family history of breast cancer, menopausal status, number of pregnancies, hormonal replacement therapy, body mass index, smoking habit, occupation, childcare responsibilities, family size

^b^Final model variables: sedentary behaviour, age, family history of breast cancer, number of pregnancies, hormonal replacement therapy, body mass index, smoking habit, occupation, childcare responsibilities, family size

^c^Final model considering tumour phenotype (HR+ vs. no cancer) variables: sedentary behaviour, age, family history of breast cancer, menopausal status, number of pregnancies, hormonal replacement therapy, body mass index, smoking habit, occupation, childcare responsibilities, family size

Abbreviations: h/d (hours per day); BC (breast cancer); HR+ (oestrogen receptor-positive, and/or progesterone receptor-positive and HER2 negative)

In postmenopausal women, those with low educational level who spend more than 2 h/d sitting showed an increased BC risk with statistically significant differences (>2-≤3 h/d: OR = 2.12 (1.28–3.62), p-value = 0.004, >5 h/d: OR = 1.75 (1.01–3.11), p-value = 0.049). In HR+ BC, this association was observed only in those with a low educational level who spent >2-≤3 h/d sitting OR = 2.15 (1.22–3.99), p-value = 0.011) (Table 3).

In the subsample of women with one or more live births, for those with low educational level, all sedentary behaviour categories of more than 2h/d sitting are statistically significantly associated with a higher risk of BC (>2-≤3 h/d: OR = 1.99 (1.20–3.43), p-value = 0.010, >3-≤5 h/d: OR = 1.67 (1.00–2.90), p-value = 0.057, >5 h/d: OR = 1.77 (1.02–3.14), p-value = 0.045) (Table 4). Additional information of other explanatory variables results is provided in S2 Table. This statistically significantly relationship was also observed in postmenopausal women for all sedentary behaviour categories (>2-≤3 h/d: OR = 2.22 (1.30–3.96), p-value = 0.005, >3-≤5 h/d: OR = 1.85 (1.07–3.32), p-value = 0.032, >5 h/d: OR = 1.30 (1.06–3.50), p-value = 0.001) and in HR+ BC, only for >2- ≤3 h/d category (OR = 2.10 (1.16–3.99), p-value = 0.017) (Table 4).

**Table 4:** Relationship between sedentary behaviour and breast cancer risk in the subsample of women with one or more live births. Final model stratifying by educational level in the whole subsample, in postmenopausal women and considering HR+ breast cancer.

			Educational level		
	Sedentary behaviour (h/d sitting)	Low OR (CI)	p value	Medium-High OR (CI)	p value

**Whole subsample** ^**a**^					
	≤2	1		1	
	>2-≤3	1.99 (1.20–3.43)	0.010	1.06 (0.73–1.53)	0.771
	>3-≤5	1.67 (1.00–2.90)	0.057	1.01 (0.69–1.50)	0.950
	>5	1.77 (1.02–3.14)	0.045	0.95 (0.63–1.41)	0.781
**Postmenopausal women** ^**b**^					
	≤2	1		1	
	>2-≤3	2.22 (1.30–3.96)	0.005	0.95 (0.60–1.49)	0.815
	>3-≤5	1.85 (1.07–3.32)	0.032	0.88 (0.55–1.41)	0.596
	>5	1.90 (1.06–3.50)	0.001	0.81 (0.49–1.33)	0.410
**HR+ BC** ^**c**^					
	≤2	1		1	
	>2-≤3	2.10 (1.16–3.99)	0.017	1.03 (0.66–1.63)	0.883
	>3-≤5	1.53 (0.82–2.95)	0.189	0.91 (0.56–1.48)	0.711
	>5	1.61 (0.83–3.19)	0.165	0.74 (0.44–1.23)	0.240

^**a**^ Final model with the variables: sedentary behaviour, age, family history of BC, menopausal status, number of pregnancies, months of breastfeeding, hormonal replacement therapy, body mass index, smoking habit, occupation, childcare responsibilities, family size

^**b**^ Final model with the variables: sedentary behaviour, age, family history of BC, number of pregnancies, months of breastfeeding, hormonal replacement therapy, body mass index, smoking habit, occupation, childcare responsibilities, family size

^**c**^ Final model considering tumour phenotype (HR+ vs. no cancer) with the variables: sedentary behaviour, age, family history of BC, menopausal status, number of pregnancies, months of breastfeeding, hormonal replacement therapy, body mass index, smoking habit, occupation, childcare responsibilities, family size

Abbreviations: h/d (hours per day); BC (breast cancer); HR+ (oestrogen receptor-positive, and/or progesterone receptor-positive and HER2 negative)

## Discussion

According to the results, sedentary behaviour is associated with an increased risk of BC when sitting for more than 2 hours per day. This relationship was only found among women with a low educational level, being also observed in postmenopausal women, in HR+ BC and in women with one or more live births.

In this study, the relationship between sedentary behaviour and BC risk has been observed taking into account evidence-based BC risk factors such as age, family history of BC, hormonal and reproductive characteristics, lifestyle behaviours and socioeoconomic and gender determinants. Currently, there is limited consensus regarding this relationship. While some studies, such as Huerta et al. [19], find an association, others, like the study conducted by Nomura et al. [15], do not observe it.

The novelty of this study lies in the observation that sedentary behaviour is associated with BC risk when studied in interaction with educational level. The results indicate that sitting more than 2 h/d increases BC risk in women with a low educational level. In the subsample of women with one or more live births, where the breastfeeding months, a well-known protective factor against BC [10], have been taken into account, this relationship has been observed in all sedentary behaviour categories. This differential impact on BC risk based on educational level is observed in other risk behaviours. For example, Bjerkaas et al. [36] found that smoking habit increases BC risk only in women with low educational level. These results suggest a possible hypothesis: the differential impact of sedentary behaviour on BC risk according to educational level could be partly explained by the profile of health-related behaviours specific to each population group. On one hand, it is known that women with high educational level have higher risk of BC [27–29] and this has been explained by their major exposition to reproductive and hormonal risk factors. Well known BC risk factors, such as nulliparity, HRT use, and fewer months of breastfeeding [20–23], are more common in women with high educational level [30, 31]. This could explain why, in our study, sedentary behaviour is not associated with BC in women with high educational level. On the other hand, in women with a low educational level, who have a lower prevalence of reproductive and hormonal risk factors, sedentary behaviour is associated with BC risk. Further research is needed to accept, reject or reformulate this hypothesis.

Regarding women’s menopausal status, studies such as those by Chan et al. [18] and Huerta et al. [19] observe that, sedentary behaviour has a greater impact on BC risk in postmenopausal women, in line with our results. In postmenopausal women, greater amounts of free oestrogens, which have previously been associated with a higher BC risk, are produced in adipose tissue [17, 37]. Thus, this could explain why sedentary behaviour, which in turn entails a higher BMI and a larger quantity of adipose tissue, are related to an increased risk of BC in postmenopausal women. Additionally, the results of the present study show that, for postmenopausal women, only those with low educational level sitting for more than 2 h/d are at a higher risk of BC, being this fact also observed in those women with one or more live births. This difference may, once again, be attributed to differences by educational level in the prevalence of hormonal and reproductive risk factors, along with the inherent hormonal changes caused by menopause.

Currently, there is still little consensus regarding the influence of sedentary behaviour on BC risk according to phenotype [11]. Our results suggest that sedentary behaviour has a greater impact on BC with hormonal receptors (HR+), which concurs with some of the current literature [19]. This association may be due to the impact of physical activity and sedentary behaviour on hormonal factors [17, 18]. Sedentary behaviour could increase the bioavailability of free oestrogens, which are known to play an important role in the development of HR+ phenotype BC [38]. Furthermore, screen-detected BC is influenced by hormonal factors, reflected in the higher diagnosis rate of HR+ tumours [39]. Therefore, the fact that the cancers included in this study are screen-detected could influence the results. But additionally, in this study’s results, the impact of sedentary behaviour on the risk of HR+ BC is observed only in women with low educational level. Once again, this may indicate the differential risk of BC based on this social determinant.

Considering other factors related to BC, this study reinforces the role of increased age, family history, nulliparity, a high BMI, and smoking habits in BC risk [20, 21, 25, 26, 40], which is widely evidenced in current literature. This underscores the results obtained regarding the role of sedentary behaviour in BC risk, as even when accounting for other known BC risk factors, sedentary behaviour also seems to influence the occurrence of this disease.

This study analysed the cancers detected in women who participated in the VR-BCSP. Some studies have observed that women participating in screening programs tend to have healthier behaviours [41, 42]. On the other hand, other studies have shown that BC most frequently diagnosed in screening are HR+ [39]. These circumstances could influence the characteristics of our sample. Nevertheless, considering that the VR-BCSP is a population-based programme targeting all women aged 45–69 with a 73% participation rate [43], the results could be extrapolated to the general population.

One limitation of this study is that the relationship between sedentary behaviour and BC in women with TN cancer could not be analysed due to the scarce sample (n = 20). Despite this, the study provides relevant information regarding the relationship with HR+ cancers. Furthermore, another limitation is that it has not been possible to analyse “live births” variable as a breast cancer risk factor because this information was not available for the entire sample.

The study data was collected through a questionnaire, which could potentially entail social desirability bias. Women may have underestimated the amount of time they spend sitting each day. In addition, the IPAQ-Short questionnaire does not differentiate between the different types of sedentary activities. Nonetheless, the IPAQ-Short is internationally validated [33], therefore lending validity to the results.

Currently, there is no consensus on the cut-off for studying sedentary behaviour and its impact on BC risk. In this study, it has been observed that spending more than 2 hours per day sitting is associated with BC risk. Other studies also observe this relationship but at different sedentary behaviour cut-offs. For example, Huerta et al. [19] show an association with more than 3 hours per day sitting, whereas Dellal et al. [12] observe it with a cut-off of more than 6 hours per day. This highlights the need to further explore this relationship.

This study’s main strength is that it provides novel results regarding the relationship between sedentary behaviour and BC while also considering health-related and socioeconomic variables such as educational level. According to the authors’ knowledge, it is one of just a few studies that consider these factors when analysing the relationship between sedentary behaviour and BC.

The European Commission Initiative on Breast Cancer (ECIBC) is currently defining a series of quality criteria that BC screening programmes must meet through the European Quality Assurance Scheme for Breast Cancer Services [44]. One of these quality criteria involves giving healthy lifestyle recommendations related to physical activity and diet during screening. Therefore, in line with ECIBC recommendations and based on the evidence revealed by this study, women who attend screening appointments should be provided with healthy lifestyle recommendations that highlight the risks of sedentary behaviour. Furthermore, the role played by sedentary behaviour in BC risk should also be considered in health promotion programmes and specific actions should be designed for women who are more likely to develop BC due to social and gender-related factors, in line with the principles of proportionate universalism.

## Conclusion

This study has revealed that sedentary behaviour is positively associated with BC risk in women with low educational level, especially in postmenopausal women and those with one or more live births. These novel results suggest that a social equity perspective should be incorporated into breast cancer prevention research and public health policies.

## Supporting information

S1 Table Relationship between sedentary behaviour and breast cancer risk for the whole sample. Final statistical model stratified by educational level. h/d (hours per day); BC (breast cancer); HRT (Hormone Replacement Therapy); BMI (Body Mass Index); FS (Family Size).
(DOCX)

S2 Table Relationship between sedentary behaviour and breast cancer risk for the subsample of women with one or more live births. Final statistical model stratified by educational level. h/d (hours per day); BC (breast cancer); HRT (Hormone Replacement Therapy); BMI (Body Mass Index); FS (Family Size).
(DOCX)

## References

[1] Sung H, Ferlay J, Siegel R, Laversanne M, Soerjomataram I, Jemal A, et al. Global Cancer Statistics 2020: GLOBOCAN Estimates of Incidence and: Mortality Worldwide for 36 Cancers in 185 Countries. *CA Cancer J Clin*. 2021; 7 (1): 209–49 doi: 10.3322/caac.21660 33538338

[2] Estimates of Cancer Incidence and Mortality in 2022, for all countries [Internet]. European Cancer Information System (ECIS). 2022. Available at: https://ecis.jrc.ec.europa.eu, accessed on 16/01/2024

[3] Anand P, Kunnumakara A, Sundaram C, Harikunar K, Tharakan S, Lai O. et al. Cancer is a Preventable Disease that Requires Major Lifestyle Changes. *Pharmaceutical Research*. 2008; 25 (9): 2097–116, doi: 10.1007/s11095-008-9661-9 18626751 PMC2515569

[4] Anderson J, Darwis N, Mackay D, Celis-Morales C, Lyall D, Sattar N, et al. Red and processed meat consumption and breast cancer: UK Biobank cohort study and meta-analysis. *Eur J Cancer*. 2018; 90: 73–82, doi: 10.1016/j.ejca.2017.11.022 29274927

[5] Godinho-Mota J, Gonçalves LV, Mota JF, Ribeiro L, Machado R, Martins K et al. Sedentary Behavior and Alcohol Consumption Increase Breast Cancer Risk Regardless of Menopausal Status: A Case-Control Study. *Nutrients*. 2019; 11 (1871), doi: 10.3390/nu11081871 31408930 PMC6723386

[6] Jones M, Schoemaker M, Wright L, Ashworth A, Swerdlow A. Smoking and risk of breast cancer in the Generations Study cohort. *Breast Cancer Res*. 2017; 19:118, doi: 10.1186/s13058-017-0908-4 29162146 PMC5698948

[7] Godinhio-Mota J, Gonçalves L, Soares L, Mota J, Martins K, Freitas-Junior I, et al. Abdominal Adiposity and Physical Inactivity Are Positively Associated with Breast Cancer: A Case-Control Study. *BioMed Res Int*. 2018; (2018), doi: 10.1155/2018/4783710 30112392 PMC6077523

[8] WHO guidelines on physical activity and sedentary behaviour. Geneva: World Health Organization; 2020. Licence: CC BY-NC-SA 3.0 IGO.chastin S,

[9] Tremblay M, Aubert S, Barnes J, Saunders T, Carson V, Latimer-Cheung A, et al. Sedentary Behavior Research Network (SBRN)–Terminology Consensus Project process and outcome. *Int J Behav Nutr Phys Act*. 2017: 14 (75), doi: 10.1186/s12966-017-0525-8 28599680 PMC5466781

[10] Zhou Y, Zhao H, Peng C. Association of sedentary behavior with the risk of breast cancer in women: update meta-analysis of observational studies. *Ann Epidemiol*. 2015, doi: 10.1016/j.annepidem.2015.05.007 26099193

[11] Nomura S, Dash C, Rosenberg L, Palmer J, Adams-Campbell LL. Sedentary time and breast cancer incidence in African American women. *Cancer Causes Control*. 2016; 27 (10): 1239–252, doi: 10.1007/s10552-016-0803-9 27632430 PMC5527706

[12] Dellal C, Brinton L, Matthews C, Lissowska J, Peplonska B, Hartman T et al. Accelerometer-based measures of active and sedentary behavior in relation to breast cancer risk. *Breast Cancer Res Treat*. 2012; 134 (3): 1279–290, doi: 10.1007/s10549-012-2129-y 22752209 PMC3534981

[13] Wiseman A, Lynch B, Cameron A, Dunstan D. Associations of change in television viewing time with biomarkers of postmenopausal breast cancer risk: the Australian Diabetes, Obesity and Lifestyle Study. *Cancer Causes Control*. 2014; 25: 1309–319, doi: 10.1007/s10552-014-0433-z 25053405

[14] Boyle T, Fritschi L, Kobayashi L, Heyworth J, Lee D, Si S, et al. Sedentary work and the risk of breast cancer in premenopausal and postmenopausal women: a pooled analysis of two case–control studies. *Occup Envir Med*. 2016; 37: 735–41, doi: 10.1136/oemed-2015-103537 27540104

[15] Nomura S, Dash C, Sheppard V, Bowen D, Allison M, Barrington W, et al. Sedentary Time and Postmenopausal Breast Cancer Incidence. *Cancer Causes Control*. 2017; 28 (12): 1405–416, doi: 10.1007/s10552-017-0968-x 28975422 PMC5687985

[16] Friedenreich C, Ryder-Burbidge C, McNeil J. Physical activity, obesity and sedentary behavior in cancer etiology: epidemiologic evidence and biologic mechanisms. *Mol Oncol*. 2021; 15: 790–800, doi: 10.1002/1878-0261.12772 32741068 PMC7931121

[17] de Boer MC, Worner EA, Verlaan D, Van Leeuwen PAM. The mechanisms and effects of physical activity on breast cancer. *Clin Breast Cancer*. 2017, doi: 10.1016/j.clbc.2017.01.006 28233686

[18] Chan D, Abar L, Cariolou M, Nanu N, Greenwood D, Bandera E et al. World Cancer Research Fund International: Continuous Update Project—systematic literature review and meta‑analysis of observational cohort studies on physical activity, sedentary behavior, adiposity, and weight change and breast cancer risk. *Cancer Causes Control*. 2019; 30: 1183–200, doi: 10.1007/s10552-019-01223-w 31471762

[19] Huerta JM, Molina AJ, Chirlaque MD, Yepes P, Moratalla-Navarro F, Moreno V, et al. Domain‑specific patterns of physical activity and risk of breast cancer sub‑types in the MCC‑Spain study. *Breast Cancer Res Treat*. 2019; 177 (3): 749–60, doi: 10.1007/s10549-019-05358-x 31317349

[20] Khalis M, Charbotel B, Chajès V, Rinaldi S, Moskal A, Biessy C, et al. Menstrual and reproductive factors and risk of breast cancer: A case-control study in the Fez region, Morocco. *Plos One*. 2018: 13(1): e0191333, doi: 10.1371/journal.pone.0191333 29338058 PMC5770054

[21] Li H, Sun X, Miller E, Wang Q, Tao P, Liu L, et al. BMI, reproductive factors, and breast cancer molecular subtypes: A case-control study and meta-analysis. Journal of Epidemiology. *J Epidemiol*. 2017; 27: 143–51, doi: 10.1016/j.je.2016.05.002 28142040 PMC5376312

[22] Nelson H, Zakher B, Cantor A, Fu R, Griffin J, O’Meara E. Risk Factors for Breast Cancer for Women Age 40 to 49: A Systematic Review and Meta-analysis. *Ann Intern Med*. 2012; 156 (9):635–648, doi: 10.1059/0003-4819-156-9-201205010-00006 22547473 PMC3561467

[23] Vinogradova Y, Coupland C, Hippisley-Cox J. Use of hormone replacement therapy and risk of breast cancer: nested case-control studies using the QResearch and CPRD databases. *BMJ*. 2020; 371: m3873, doi: 10.1136/bmj.m3873 33115755 PMC7592147

[24] O’Donoghe G, Perchoux C, Mensah K, Lakerveld J, van der Ploeg H, Bernaards C, et al. A systematic review of correlates of sedentary behaviour in adults aged 18–65 years: a socio-ecological approach. *BMC Public Health*. 2016; 16 (163), doi: 10.1186/s12889-016-2841-3 26887323 PMC4756464

[25] Macacu A, Autier P, Boniol M, Boyle P. Active and passive smoking and risk of breast cancer: a meta-analysis. *Breast Cancer Res Treat*. 2015; 154(2): 213–24, doi: 10.1007/s10549-015-3628-4 26546245

[26] Maliniak M, Gapstur S, McCullough L, Rees-Punia E, Gaudet MM, Um CY, et al. Joint associations of physical activity and body mass index with the risk of established excess body fatness‑related cancers among postmenopausal women. *Cancer Causes Control*. 2020; 32(2):127–138, doi: 10.1007/s10552-020-01365-2 33185805

[27] Dinkel D, Hein N, Snyder K, Siahpush M, Maloney S, Smith L, et al. The impact of body mass index and sociodemographic factors on moderate to vigorous physical activity and sedentary behaviors of women with young children: A cross-sectional examination. *Women’s Health*. 2020; 16: 1–9, doi: 10.1177/1745506519897826 31971094 PMC6984422

[28] Berger E, Maitre N, Mancini FR, Baglietto L, Perduca V, Colineaux H, et al. The impact of lifecourse socio-economic position and individual social mobility on breast cancer risk. *BMC Cancer*. 2020; 20: 1138 doi: 10.1186/s12885-020-07648-w 33228587 PMC7684912

[29] Fujino Y, Mori M, Tamakoshi A, Sakauchi F, Suzuki S, Wakai K, et al. A prospective study of educational background and breast cancer among Japanese women. *Cancer Causes Control*. 2009; 19:931–37, doi: 10.1007/s10552-008-9154-5 18389378

[30] Butt Z, Furqan S, Arif S, Raza M, Ashfaq U, Shahbaz U. Breast cancer risk factors: A comparison between pre-menopausal and post-menopausal women. *J Pak Med Assoc*. 2012; 62 (2): 120–24 22755371

[31] Du Y, Melchert HU, Schäfer-Korting M. Hormone replacement therapy in Germany: Determinants and possible health-related outcomes. Results of National Health Surveys from 1984 to 1999. *Maturitas*. 2005; 52: 223–34, doi: 10.1016/j.maturitas.2005.01.014 16040212

[32] Pinto-Carbó M, Vanaclocha-Espí M, Ibañez J, Hernández-García M, Salas D, Molina-Barceló A. Analysis of sedentariness in women from a gender and equity perspective. *Eur J Sport Sci*. 2021; 22 (12): 1898–907, doi: 10.1080/17461391.2021.1975829 34463206

[33] Johnsson A, Broberg P, Johnsson A, Tornberg A, Olsson H. Occupational sedentariness and breast cancer risk. *Acta Oncol*. 2016; 56 (1): 75–80, doi: 10.1080/0284186X.2016.1262547 27919198

[34] Lee J, Lee J, Lee DW, Kim HR, Kang M. Sedentary work and breast cancer risk: A systematic review and meta-analysis. *J Occup Health*. 2021; 63: e12239, doi: 10.1002/1348-9585.12239 34161650 PMC8221371

[35] Rosenberg D, Bull F, Marshall A, Sallis J, Bauman A. Assessment of Sedentary Behavior With the International Physical Activity Questionnaire. *JPAH*. 2008; 5 (1): 30–44. doi: 10.1123/jpah.5.s1.s30 18364524

[36] Bjerkaas E, Parajuli R, Engeland A, Maskarinez G, Weiderpass E, Torhild Gram I. Social inequalities and smoking-associated breast cancer—Results from a prospective cohort study. *Prev Med*. 2015; 73: 125–29, doi: 10.1016/j.ypmed.2015.01.004 25620729

[37] Oh H, Arem H, Matthews CE, Wentzensen N, Reding KW, Brinton LA, et al. Sitting, physical activity, and serum oestrogen metabolism in postmenopausal women: the Women’s Health Initiative Observational Study. *BJC*. 2017; 117: 1070–78, doi: 10.1038/bjc.2017.268 28817836 PMC5625673

[38] Ellingjord-Dale M, Vos L. Tretli S, Hofvind S, dos-Santos-Silva I, Ursin G. Parity, hormones and breast cancer subtypes—results from a large nested case-control study in a national screening program. *Breast Cancer Res*. 2017; 19 (10), doi: 10.1186/s13058-016-0798-x 28114999 PMC5259848

[39] Hernández-García M, Molina-Barceló A, Vanaclocha-Espí M, Zurriaga Ó, Pérez-Gómez B, Aragonés N, et al. Diferences in breast cancer-risk factors between screen-detected and non-screen-detected cases (MCC-Spain study). *Cancer Causes Control*. 2022; 33:125–36, doi: 10.1007/s10552-021-01511-4 34817770 PMC8739309

[40] World Cancer Research Fund/American Institute for Cancer Research. Continuous Update Project Expert Report 2018. Diet, nutrition, physical activity and breast cancer. Available at dietandcancerreport.org

[41] Portero de la Cruz S; Béjar LM; Cebrino J. Temporal Evolution and Associated Factors of Adherence to Mammography Screening among Women in Spain: Results from Two National Health Surveys (2017–2020). *Healthcare*. 2023; 11, 2934, doi: 10.3390/healthcare11222934 37998426 PMC10671473

[42] Patrão AL, de Almeida MDCC, Matos SMA, Menezes G, Gabrielli L, Goes EF, et al. Healthy lifestyle behaviors and the periodicity of mammography screening in Brazilian women. *Womens Health*. 2021; 17:17455065211063294, doi: 10.1177/17455065211063294 34841999 PMC8640279

[43] Molina-Barceló A, Moreno Salas J, Peiró-Pérez R, Arroyo G, Ibáñez Cabanell J, Vanaclocha Espí M, et al. desigualdades de acceso a los programas de cribado del cáncer en España y cómo reducirlas: datos de 2013 y 2020. Rev Esp Salud Pública. 2021; 29 33496270

[44] Janusch-Roi A, Neamtiu L, Dimitrova N, Ulutürk A, García-Escribano M, Sardanelli M, et al., European Commission Initiative on Breast Cancer–Manual for Breast Cancer Services–European Quality Assurance Scheme for Breast Cancer Services, EUR 30750 EN, Publications Office of the European Union, Luxembourg, 2021, ISBN 978-92-76-39192-0, doi: 10.2760/155701, JRC125431

