# Supplementary material for: Interaction of sedentary behaviour and educational level in breast cancer risk
Source: PLoS One. 2024 May 16;19(5):e0300349. doi: 10.1371/journal.pone.0300349 (PMC11098410; doi:10.1371/journal.pone.0300349)
Supplement: S1 Table — Final statistical model stratified by educational level. h/d (hours per day); BC (breast cancer); HRT (Hormone Replacement Therapy); BMI (Body Mass Index); FS (Family Size). (DOCX) [file pone.0300349.s001.docx]

**S1Table. Relationship between sedentary behaviour and breast cancer risk for the whole sample. Final statistical model stratified by educational level.**

|  |  | **Educational level** | |
| --- | --- | --- | --- |
|  |  | **Low** | **Medium-High** |
|  |  | OR (CI) | OR (CI) |
|  | **Sedentary behaviour**  **(h/d sitting)** |  |  |
|  | ≤2 | 1 | 1 |
|  | >2-≤3 | 1.93 (1.19-3.21) | 0.96 (0.68-1.35) |
|  | >3- ≤5 | 1.51 (0.92-2.54) | 1.00 (0.70-1.43) |
|  | >5 | 1.66 (0.98-2.86) | 1.01 (0.71-1.44) |
| **Adjustment** | **Age (years)** | 1.07 (1.03-1.12) | 1.03 (1.00-1.06) |
|  | **BC family history** |  |  |
|  | No | 1 | 1 |
|  | Yes | 1.23 (0.86-1.74) | 1.34 (1.04-1.73) |
| **Hormonal/**  **Reproductive** | **No of pregnancies** |  |  |
|  | ≥2 | 1 | 1 |
|  | 1 | 2.43 (1.59-3.63) | 1.31 (0.96-1.78) |
|  | 0 | 0.85 (0.33-1.80) | 1.39 (0.96-2.00) |
|  | **Menopausal status** |  |  |
|  | Premenopause | 1 | 1 |
|  | Postmenopause | 1.10 (0.49-2.84) | 0.79 (0.56-1.11) |
|  | **HRT** |  |  |
|  | No | 1 | 1 |
|  | Yes | 1.44 (0.82-2.36) | 1.19 (0.74-1.83) |
| **Lifestyles** | **BMI** | 1.03 (0.99-1.06) | 1.03 (1.00-1.05) |
|  | **Smoking habit** |  |  |
|  | Non-smoker | 1 | 1 |
|  | Current | 1.50 (0.97-2.26) | 1.29 (0.96-1.73) |
|  | Former | 1.39 (0.90-2.10) | 1.45 (1.08-1.93) |
| **Socioeconomic** | **Occupation** |  |  |
|  | Manual | 1 | 1 |
|  | Non manual | 1.64 (0.74-3.26) | 0.99 (0.72-1.36) |
|  | Non-working | 0.59 (0.30-1.09) | 1.16 (0.74-1.77) |
|  | Homemaker | 1.04 (0.70-1.56) | 1.09 (0.78-1.53) |
| **Gender** | **Childcare responsibilities** |  |  |
|  | No | 1 | 1 |
|  | Yes | 1.01 (0.58-1.67) | 0.82 (0.58-1.14) |
|  | **FS** |  |  |
|  | Small | 1 | 1 |
|  | Medium-Large | 0.95 (0.66-1.36) | 0.88 (0.67-1.17) |

Abbreviations: h/d (hours per day); BC (breast cancer); HRT (Hormone Replacement Therapy); BMI (Body Mass Index); FS (Family Size)
