# Supplementary material for: Interaction of sedentary behaviour and educational level in breast cancer risk
Source: PLoS One. 2024 May 16;19(5):e0300349. doi: 10.1371/journal.pone.0300349 (PMC11098410; doi:10.1371/journal.pone.0300349)
Supplement: S2 Table — Final statistical model stratified by educational level. h/d (hours per day); BC (breast cancer); HRT (Hormone Replacement Therapy); BMI (Body Mass Index); FS (Family Size). (DOCX) [file pone.0300349.s002.docx]

**S2 Table. Relationship between sedentary behaviour and breast cancer risk for the subsample of women with one or more live births. Final statistical model stratified by educational level.**

|  |  | **Educational level** | |
| --- | --- | --- | --- |
|  |  | **Low**  OR (CI) | **Medium-High**  OR (CI) |
|  |  |  |  |
|  | **Sedentary behaviour**  **(h/d sitting)** |  |  |
|  | ≤2 | 1 | 1 |
|  | >2-≤3 | 1.99 (1.20-3.43) | 1.06 (0.69-1.53) |
|  | >3-≤5 | 1.67 (1.00-2.90) | 1.01 (0.69-1.50) |
|  | >5 | 1.77 (1.02-3.14) | 0.95 (0.63-1.41) |
| **Adjustment** | **Age (years)** | 1.07 (1.03-1.12) | 1.02 (0.99-1.05) |
|  | **BC family history** |  |  |
|  | No | 1 | 1 |
|  | Yes | 1.20 (0.82-1.73) | 1.36 (1.01-1.80) |
| **Hormonal/**  **Reproductive** | **No of pregnancies** |  |  |
|  | ≥2 | 1 | 1 |
|  | 1 | 2.49 (1.59-3.80) | 1.29 (0.92-1.77) |
|  | **Months of breastfeeding** |  |  |
|  | ≥6 | 1 | 1 |
|  | <6 | 0.95 (0.62-1.44) | 0.93 (0.66-1.30) |
|  | 0 | 1.06 (0.69-1.60) | 1.34 (0.96-1.85) |
|  | **Menopausal status** |  |  |
|  | Premenopause | 1 | 1 |
|  | Postmenopáusica | 0.90 (0.39-2.36) | 0.86 (0.58-1.27) |
|  | **HRT** |  |  |
|  | No | 1 | 1 |
|  | Yes | 1.56 (0.88-2.57) | 1.32 (0.79-2.07) |
| **Lifestyles** | **BMI** | 1.03 (1.00-1.06) | 1.03 (1.00-1.06) |
|  | **Smoking habit** |  |  |
|  | Non-smoker | 1 | 1 |
|  | Current | 1.48 (0.94-2.29) | 1.32 (0.95-1.82) |
|  | Former | 1.48 (0.94-2.25) | 1.33 (0.96-1.85) |
| **Socioeconomic** | **Occupation** |  |  |
|  | Manual | 1 | 1 |
|  | Non manual | 1.90 (0.85-3.83) | 1.01 (0.71-1.45) |
|  | Non-working | 0.62 (0.30-1.20) | 1.24 (0.76-1.97) |
|  | Homemaker | 1.08 (0.71-1.67) | 1.10 (0.76-1.58) |
| **Gender** | **Childcare responsibilities** |  |  |
|  | No | 1 | 1 |
|  | Yes | 1.07 (0.61-1.37) | 0.86 (0.60-1.22) |
|  | **FS** |  |  |
|  | Small | 1 | 1 |
|  | Medium-Large | 0.94 (0.65-1.37) | 0.83 (0.61-1.12) |

Abbreviations: h/d (hours per day); BC (breast cancer); HRT (Hormone Replacement Therapy); BMI (Body Mass Index); FS (Family Size)
